# Occam’s Quantum Strop: Synchronizing and Compressing Classical Cryptic Processes via a Quantum Channel

**DOI:** 10.1038/srep20495

**Published:** 2016-02-15

**Authors:** John R. Mahoney, Cina Aghamohammadi, James P. Crutchfield

**Affiliations:** 1 Complexity Sciences Center and Department of Physics, University of California at Davis, One Shields Avenue, Davis, CA 95616, USA.

## Abstract

A stochastic process’ statistical complexity stands out as a fundamental property: the minimum information required to synchronize one process generator to another. How much information is required, though, when synchronizing over a quantum channel? Recent work demonstrated that representing causal similarity as quantum state-indistinguishability provides a quantum advantage. We generalize this to synchronization and offer a sequence of constructions that exploit extended causal structures, finding substantial increase of the quantum advantage. We demonstrate that maximum compression is determined by the process’ cryptic order–a classical, topological property closely allied to Markov order, itself a measure of historical dependence. We introduce an efficient algorithm that computes the quantum advantage and close noting that the advantage comes at a cost–one trades off prediction for generation complexity.

Discovering and describing correlation and pattern are critical to progress in the physical sciences. Observing the weather in California last Summer we find a long series of sunny days interrupted only rarely by rain–a pattern now all too familiar to residents. Analogously, a one-dimensional spin system in a magnetic field might have most of its spins “up” with just a few “down”–defects determined by the details of spin coupling and thermal fluctuations. Though nominally the same pattern, the domains of these systems span the macroscopic to the microscopic, the multi-layer to the pure. Despite the gap, can we meaningfully compare these two patterns?

To exist on an equal descriptive footing, they must each be abstracted from their physical embodiment by, for example, expressing their generating mechanisms via minimal probabilistic encodings. Measures of unpredictability, memory, and structure then naturally arise as information-theoretic properties of these encodings. Indeed, the fundamental interpretation of (Shannon) information is as a rate of encoding such sequences. This recasts the informational properties as answers to distinct communication problems. For instance, a process’ structure becomes the problem of two observers, Alice and Bob, synchronizing their predictions of the process.

However, what if the communication between Alice and Bob is not classical? What if Alice instead sends qubits to Bob–that is, they synchronize over a quantum channel? Does this change the communication requirements? More generally, does quantum communication enhance our understanding of what “pattern” is in the first place? What if the original process is itself quantum? More practically, is the quantum encoding more compact?

A provocative answer to the last question appeared recently^1^,^2^,^3^ suggesting that a quantum representation can compress a stochastic process beyond its known classical limits^4^. In the following, we introduce a new construction for quantum channels that improves and broadens that result to any memoryful stochastic process, is highly computationally efficient, and points toward optimal quantum compression. Importantly, we draw out the connection between quantum compressibility and process cryptic order–a purely classical property that was only recently discovered^5^. Finally, we discuss the subtle way in which the quantum framing of pattern and structure differs from the classical.

## Synchronizing Classical Processes

To frame these questions precisely, we focus on patterns generated by discrete-valued, discrete-time stationary stochastic processes. There is a broad literature that addresses such emergent patterns^6^,^7^,^8^. In particular, computational mechanics is a well-developed theory of pattern whose primary construct–the 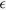-*machine*–is a process’ minimal, unifilar predictor^4^. The 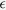-machine’s *causal states* 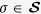 are defined by the equivalence relation that groups all histories 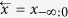 that lead to the same prediction of the future 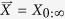:


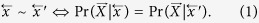


A process’ 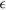-machine allows one to directly calculate its measures of unpredictability, memory, and structure.

For example, the most basic question about unpredictability is, how much uncertainty about the next future observation remains given complete knowledge of the infinite past? This is measured by the well-known Shannon *entropy rate h*_*μ*_^9^,^10^,^11^,^12^:


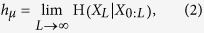


where *X*_*L*_ denotes the symbol random variable (r.v.) at time *L*, *X*_0:*L*_ denotes the length-*L* block of symbol r.v.s *X*_0_, …, *X*_*L*−1_, and 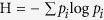 is the Shannon entropy (in bits using log base 2) of the probability distribution {*p*_*i*_}^13^. A process’ 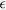-machine allows us to directly calculate this in closed form as the state-averaged branching uncertainty:


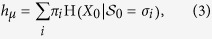


where *π_i_* denotes the stationary distribution over the causal states. This form is possible due to the 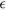-machine’s *unifilarity*: in each state *σ*, each symbol *x* leads to at most one successor state *σ*′.

One can ask the complementary question, given knowledge of the infinite past, how much can we reduce our uncertainty about the future? This quantity is the mutual information between the past and future and is known the *excess entropy*^9^:


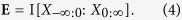


It is the total amount of future information *predictable* from the past. Using the 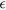-machine we can directly calculate it also:


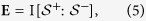


where 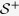 and 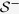 are the forward (predictive) and reverse (retrodictive) causal states, respectively^5^. This suggests we think of any process as channel that communicates the past to the future through the present. In this view **E** is the information transmission rate through the present “channel”. The excess entropy has been applied to capture the total predictable information in such diverse systems as Ising spin models^14^, diffusion in nonlinear potentials^15^, neural spike trains^16^,^17^,^18^, and human language^19^.

What memory is necessary to *implement* predicting **E** bits of the future given the past? Said differently, what resources are required to instantiate this putative channel? Most basically, this is simply the historical information the process remembers and stores in the present. The minimum necessary such information is that stored in the causal states, the *statistical complexity*^4^:


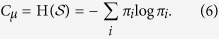


Importantly, it is lower-bounded by the excess entropy:


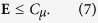


What do these quantities tell us? Perhaps the most surprising observation is that there is a large class of *cryptic processes* for which **E** ≪ *C*_*μ*_^5^. The structural mechanism behind this difference is characterized by the *cryptic order*: the minimum *k* for which 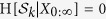. A related and more familiar property is the *Markov order*: the smallest *R* for which 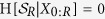. Markov order reflects a process’ historical dependence. These orders are independent apart from the fact that *k* ≤ *R*^20^,^21^. It is worth pointing out that the equality **E** = *C*_*μ*_ is obtained exactly for cryptic order *k* = 0 and, furthermore, that this corresponds with *counifilarity*–for each state *σ*′ and each symbol *x*, there is at most one prior state *σ* that leads to *σ*′ on a transition generating *x*^21^.

These properties play a key role in the following communication scenario where we have a given process’ 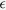-machine in hand. Alice and Bob each have a copy. Since she has been following the process for some time, using her 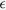-machine Alice knows that the process is currently in state *σ*_*j*_, say. From this knowledge, she can use her 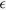-machine to make the optimal probabilistic prediction 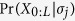 about the process’ future (and do so over arbitrarily long horizons *L*). While Bob is able to produce all such predictions from each of his 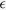-machine’s states, he does not know which particular state is currently relevant to Alice. We say that Bob and Alice are *unsynchronized*.

To communicate the relevant state to Bob, Alice must send at least *C*_*μ*_ bits of information. More precisely, to communicate this information for an ensemble (size N → ∞) of 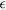-machines, she may, by the Shannon noiseless coding theorem^13^, send *NC*_*μ*_ bits. Under this interpretation, *C*_*μ*_ is a fundamental measure of a process’ structure in that it characterizes not only the correlation between past and future, but also the *mechanism* of prediction. In the scenario with Alice and Bob, *C*_*μ*_ is seen as the communication cost to synchronize. We can also imagine Alice using this channel to communicate with her future self. In this light, *C*_*μ*_ is understood as a fundamental measure of a process’ internal memory.

## Results

### Quantum Synchronization

What if Alice can send *qubits* to Bob? Consider a communication protocol in which Alice encodes the causal state in a quantum state that is sent to Bob. Bob then extracts the information through measurement of this quantum state. Their communication is implemented via a quantum object–the *q-machine* –that simulates the original stochastic process. It sports a single parameter that sets the horizon-length *L* of future words incorporated in the quantum-state superpositions it employs. We monitor the q-machine protocol’s efficacy by comparing the quantum-state information transmission rate to the classical causal-state rate (*C*_*μ*_).

The q-machine *M*(*L*) consists of a set 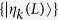 of pure *signal states* that are in one-to-one correspondence with the classical causal states 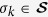. Each signal state 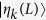 encodes the set of length-*L* words that may follow *σ*_*k*_, as well as each corresponding conditional probability used for prediction from *σ*_*k*_. Fixing *L*, we construct quantum states of the form:


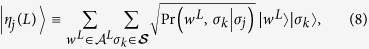


where *w*^*L*^ denotes a length-*L* word and 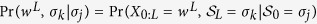. Due to 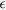-machine unifilarity, a word *w*^*L*^ following a causal state *σ*_*j*_ leads to only one subsequent causal state. Thus, 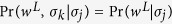. The resulting Hilbert space is the product 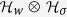. Factor space 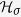 is of size 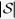, the number of classical causal states, with basis elements 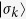. Factor space 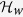 is of size 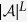, the number of length-*L* words, with basis elements 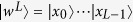.

Note that the *L* = 1 q-machine *M*(1) is equivalent to the construction introduced in ref. 1. Additionally, insight about the q-machine can be gained through its connection with the classical concatenation machine defined in ref. 22; the q-machine *M*(*L*) is equivalent to the q-machine *M*(1) derived from the *L*th concatenation machine.

Having specified the state space, we now describe how the q-machine produces symbol sequences. Given one of the pure quantum signal states, we perform a projective measurement in the 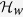 basis. This results in a symbol string 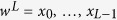, which we take as the next *L* symbols in the generated process. Since the 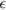-machine is unifilar, the quantum conditional state must be in some basis state 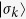 of 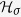. Subsequent measurement in this basis then indicates the corresponding classical causal state with no uncertainty.

Observe that the probability of a word *w*^*L*^ given quantum state 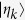 is equal to the probability of that word given the analogous classical state *σ*_*k*_. Also, the classical knowledge of the subsequent corresponding causal state can be used to prepare a subsequent quantum state for continued symbol generation. Thus, the q-machine generates the desired stochastic process and is, in this sense, equivalent to the classical 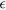-machine.

Focus now on the q-machine’s initial quantum state:


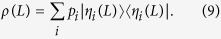


We see this mixed quantum state is composed of pure signal states combined according to the probabilities of each being prepared by Alice (or being realized by the original process that she observes). These are simply the probabilities of each corresponding classical causal state, which we take to be the stationary distribution: *p*_*i*_ = *π*_*i*_. In short, quantum state *ρ*(*L*) is what Alice must transmit to Bob for him to successfully synchronize. Later, we revisit this scenario to discuss the tradeoffs associated with the q-machine representation.

If Alice sends a large number *N* of these states, she may, according to the quantum noiseless coding theorem^23^, compress this message into *NS*(*ρ*(*L*)) qubits, where *S* is the von Neumann entropy 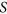
(*ρ*) = tr(*ρ* log(*ρ*)). Due to its parallel with *C*_*μ*_, and for convenience, we define the function:


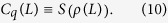


Recall that, classically, Alice must send *NC*_*μ*_ bits. To the extent that *NC*_*q*_(*L*) is smaller, the quantum protocol will be more efficient. In this particular sense, the q-machine is a compressed representation of the original process and its ε-machine.

### Example Processes: *C*_*q*_(*L*)

Let’s now draw out specific consequences of using the q-machine. We explore protocol efficiency by calculating *C*_*q*_(*L*) for several example processes, each chosen to illustrate distinct properties: the q-machine affords a quantum advantage, further compression can be found at longer horizons *L*, and the compression rate is minimized at the horizon length *k*–the cryptic order of the classical process^21^.

For each example, we examine a process family by sweeping one transition probability parameter, illustrating *C*_*q*_(*L*) and its relation to classical bounds *C*_*μ*_ and **E**. Additionally, we highlight a single representative process at one generic transition probability. Following these examples, we turn to discuss more general properties of q-machine compression that apply quite broadly and how the results alter our notion of quantum structural complexity.

#### Biased Coins Process

**Figure 1:**
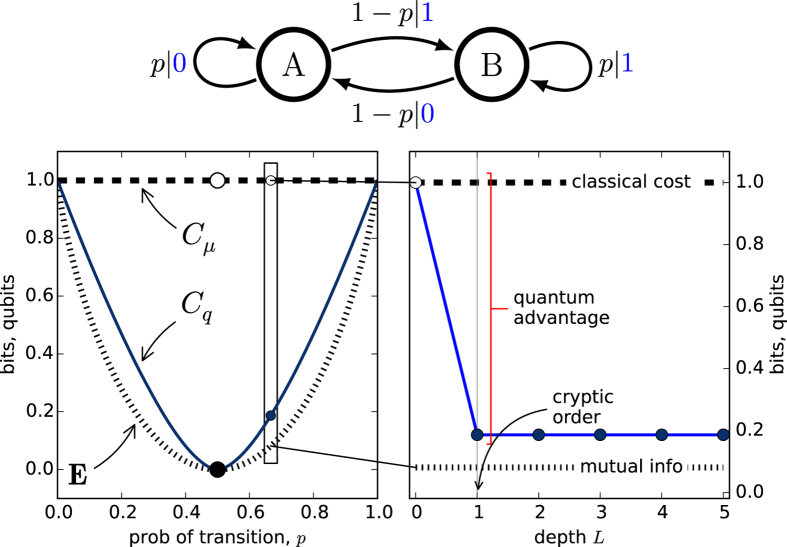
Biased Coins Process: (top) 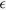-Machine. Edges are conditional probabilities. For example, self-loop label *p*|0 from state *A* indicates Pr(0|*A*) = *p*. (left) Statistical complexity *C*_*μ*_, quantum state entropy *C*_*q*_(*L*), and excess entropy **E** as a function of *A*’s self-loop probability *p* ∈ [0, 1]. *C*_*q*_(1) (dark blue) lies between *C*_*μ*_ and **E** (bits), except for extreme parameters and the center (*p* = 1/2). (right) For *p* = 0.666, *C*_*q*_(*L*) decreases from *L* = 0 to *L* = 1 and is then constant; the process is maximally compressed at *L* = 1, its cryptic order *k* = 1. This yields substantial compression: *C*_*q*_(1) ≪ *C*_*μ*_.

The Biased Coins Process provides a first, simple case that realizes a nontrivial quantum state entropy^1^. There are two biased coins, named *A* and *B*. The first generates 0 with probability *p*; the second, 0 with probability 1 − *p*. At each step, one coin is flipped–which coin is flipped depends on the result of the previous flip. If the previous flip yielded a 1, the next flip is made using coin *B*. If the previous flip yielded a 1, the next flip is made using coin *A*. Otherwise the same coin is flipped. Its two causal-state 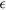-machine is shown in Fig. 1(top).

Consider p ≈ 1/2. The generated sequence is close to that of a fair coin. And, starting with coin *A* or *B* makes little difference to the future; there is little to predict about future sequences. This intuition is quantified by the predictable information **E** ≈ 0, when *p* is near 1/2. See Fig. 1(left).

In contrast, since the causal states have equal probability, *C*_*μ*_ = 1 bit independent of parameter *p*. (All information measures are quoted in log base 2.) This is because there is always *some*, albeit very little, predictive advantage to remembering whether the last symbol was 0 or 1. Retaining this advantage, however small, requires the use of an entire (classical) bit. The gap between *C*_*μ*_ and **E** presents an opportunity for large quantum improvement. It is only at the exact value *p* = 1/2 where the two causal states merge, this advantage disappears, and the process becomes memoryless or independent, identically distributed (IID). This is reflected in the discontinuity of *C*_*μ*_ as *p* → 1/2, which is sometimes misinterpreted as a deficiency of *C*_*μ*_. Contrariwise, this feature follows naturally from the equivalence relation Eq. (1) and is a signature of symmetry.

Now, let’s consider these complexities in the quantum setting where we monitor communication costs using *C*_*q*_(*L*). To understand its behavior, we first write down the q-machine’s states. For *L* = 0, we have the trivial 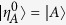 and 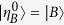. For *L* = 1, we have 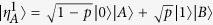 and 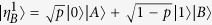. The von Neumann entropy of the former is simply the Shannon information of the signal state distribution; that is, *C*_*q*_(0) = *C*_*μ*_. In the latter, however, the two quantum states have a nonzero overlap (inner product). This implies that the von Neumann entropy is smaller than the Shannon entropy 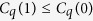. (See ref. 24 Thm. 11.10.) Also, making use of the Holevo bound, we see that **E** ≤ *C*_*q*_(1)^1^,^25^. These bounds are maintained for all *L*: **E** ≤ *C*_*q*_(*L*) ≤ *C*_*μ*_. This follows by considering the q-machine *M*(1) of the *L*th classical concatenation, described above.

(Note that for *p* ∈ {0, 1/2, 1} these quantities are all equal and equal to zero. This comes from the simplification of process topology caused by state merging dictated by the predictive equivalence relation, Eq. (1).)

How do costs change with sequence length *L*? To see this Fig. 1(right) expands the left view for a single value of *p*. As expected, *C*_*q*_(*L*) decreases from *L* = 0 to *L* = 1. However, it then remains constant for all *L* ≥ 1. There is no additional quantum state-compression afforded by expanding the q-machine to use longer horizons.

The Biased Coins Process has been analyzed earlier using a construction equivalent to an *L* = 1 q-machine^1^, similarly finding that the number of required qubits falls between **E** and *C*_*μ*_. The explanation there for this compression (*C*_*q*_(1) < *C*_*μ*_) was lack of counifilarity in the process’ 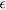-machine. More specifically, ref. 1 showed that **E** = *C*_*q*_ = *C*_*μ*_ if and only if the 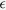-machine is counifilar, and **E** < *C*_*q*_ < *C*_*μ*_ otherwise. The Biased Coins Process is easily seen to be noncounifilar and so the inequality follows.

This previous analysis happens to be sufficient for the Biased Coins Process, since *C*_*q*_(*L*) does not decrease beyond *L* = 1. Unfortunately, only this single, two-state process was analyzed previously when, in fact, the space of processes is replete with richly structured behaviors^26^. With this in mind, and to show the power of the q-machine, we step into deeper water and consider a 7-state process that is almost periodic with a random phase-slip.

#### *R*-*k* Golden Mean Process

**Figure 2:**
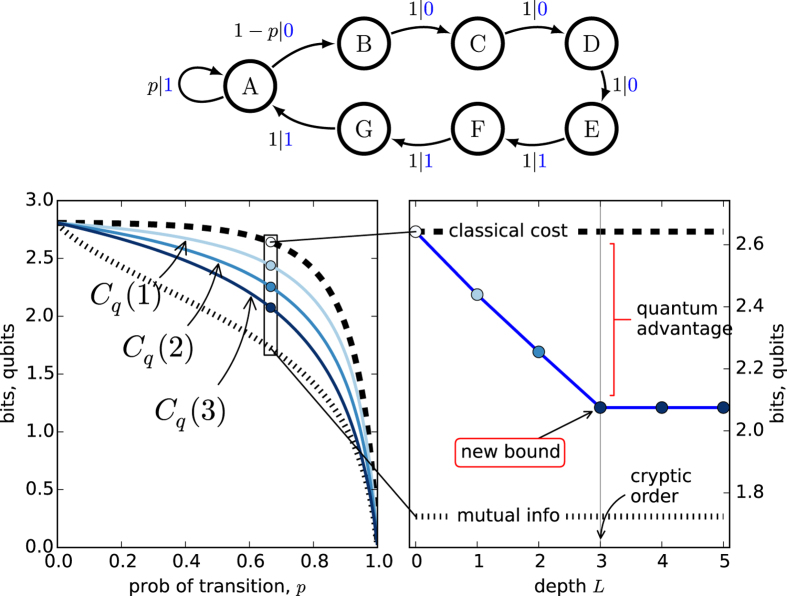
4-3 Golden Mean Process: (top) The 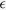-machine. (left) Statistical complexity *C*_*μ*_, quantum state entropy *C*_*q*_(*L*), and excess entropy **E** as a function of *A*’s self-loop probability *p* ∈ [0, 1]. *C*_*q*_(*L*) is calculated and plotted (light to dark blue) up to *L* = 5. (right) For *p* = 0.666, *C*_*q*_(*L*) decreases monotonically until *L* = 3–the process’ cryptic order. The additional compression is substantial: *C*_*q*_(3) ≪ *C*_*q*_(1).

The *R*-*k* Golden Mean Process is a useful generalization of the Markov order-1 Golden Mean Process that allows for the independent specification of Markov order *R* and cryptic order *k*^20^,^21^. Figure 2(top) illustrates its 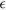-machine. We take *R* = 4 and *k* = 3.

The calculations in Fig. 2(left) show again that *C*_*q*_(*L*) generically lies between **E** and *C*_*μ*_, across this family of processes. In contrast with the previous example, *C*_*q*_(*L*) continues to decrease beyond *L* = 1. Figure 2(right) illustrates that the successive q-machines continue to reduce the von Neumann entropy: *C*_*μ*_ > *C*_*q*_(1) > *C*_*q*_(2) > *C*_*q*_(3). However, there is no further improvement beyond a future-depth of *L* = 3, the cryptic order: *C*_*q*_(3) = *C*_*q*_(*L* > 3). It is important to note that the compression improvements at stages *L* = 2 and *L* = 3 are significant. Therefore, a length-1 quantum representation misses the majority of the quantum advantage.

To understand these results we need to sort out how quantum compression stems from noncounifilarity. In short, the latter leads to quantum signal states with nonzero overlap that allow for super-classical compression. Let’s explain using the current example. There is one noncounifilar state in this process, state *A*. Both states *A* and *G* lead to *A* on a symbol 1. Due to this, at *L* = 1, the two q-machine states:


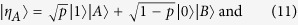



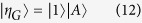


have a nonzero overlap of 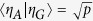. (All other overlaps in the *L* = 1 q-machine vanish.) As with the Biased Coins Process, this leads to the inequality *C*_*q*_(1) < *C*_*μ*_.

Extending the representation to *L* = 2 words, we find three nonorthogonal quantum states:


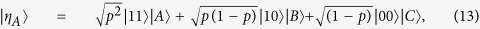



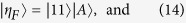



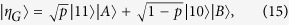


with three nonzero overlaps 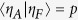, 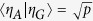, and 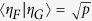.

Note that the overlap 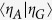 is unchanged. This is because the conditional futures are identical once the merger on symbol 1 has taken place. That is, the words 11 and 10, which contribute to the *L* = 2 overlap 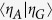, simply derive from the prefix 1, which was the source of the overlap at *L* = 1. In order to obtain a change in this or any other overlap, there must be a *new* merger-inducing prefix (for that state-pair). (See Sec. 5 for computational implications.) Since all quantum amplitudes are positive, each pairwise overlap is a nondecreasing function of *L*.

At *L* = 2 we have two such new mergers: 11 for 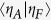 and 11 for 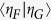. This additional increase in pairwise overlaps leads to a second decrease in the von Neumann entropy. (See Sec. 3 for details.) Then, at *L* = 3, we find three new mergers: 111 for 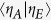, 111 for 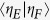, and 111 for 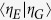. As before, the pre-existing mergers simply acquire suffixes and do not change the degree of overlap.

Importantly, we find that at *L* = 4 there are no new mergers. That is, any length-4 word that leads to the merging of two states must merge before the fourth symbol. In general, the length at which the last merger occurs is equivalent to the cryptic order^21^. Further, it is known that the von Neumann entropy is a function of pairwise overlaps of signal states^27^. Therefore, a lack of new mergers, and thus constant overlaps, implies that the von Neumann entropy is constant. This demonstrates that *C*_*q*_(*L*) is constant for *L* ≥ *k*, for *k* the cryptic order.

The *R*-*k* Golden Mean Process was selected to highlight the unique role of the cryptic order, by drawing a distinction between it and Markov order. The result emphasizes the physical significance of the cryptic order. In the example, it is not until *L* = 4 that a naive observer can synchronize to the causal state; this is shown by the Markov order. For example, the word 000 induces two states *D* and *E*. Just one more symbol synchronizes to either *E* (on 0) or *F* (on 1). Yet recall that synchronization can come about in two ways. A word may either induce a path merger or a path termination. All merger-type synchronizations must occur no later than the last termination-type synchronization. This is equivalently stated: the cryptic order is never greater than the Markov order^21^.

In the current example, we observe this termination-type of synchronization on the symbol following 000. For instance, 0000 does not lead to the merger of paths originating in multiple states. Rather, it eliminates the possibility that the original state might have been *B*.

It is the final merger-type synchronization at *L* = 3 that leads to the final unique-prefix quantum merger and, thus, to the ultimate minimization of the von Neumann entropy. So, we see that in the context of the q-machine, the most efficient state compression is accomplished at the process’ cryptic order. (One could certainly continue beyond the cryptic order, but at best this increases implementation cost with no functional benefit.)

#### Nemo Process

**Figure 3:**
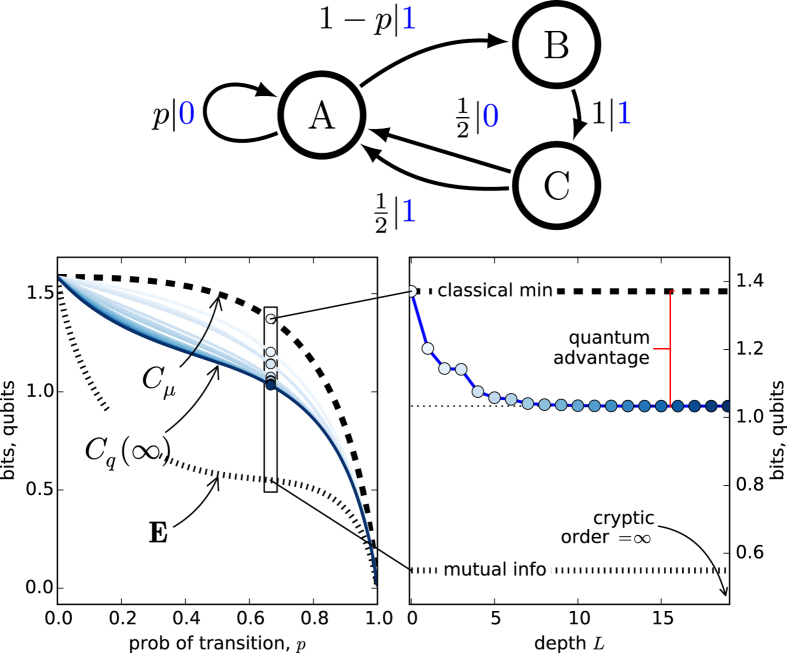
Nemo Process: (top) Its 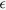-machine. (left) Statistical complexity *C*_*μ*_, quantum state entropy *C*_*q*_(*L*), and excess entropy **E** as a function of *A*’s self-loop probability *p* ∈ [0, 1]. *C*_*q*_(*L*) is calculated and plotted (light to dark blue) for *L* = 0, 1, ..., 19. (right) For *p* = 0.666, *C*_*q*_(*L*) decreases monotonically, never reaching the limit since the process’ cryptic order is infinite. The full quantum advantage is realized only in the limit.

To demonstrate the challenges in quantum compressing typical memoryful stochastic processes, we conclude our set of examples with the seemingly simple three-state Nemo Process, shown in Fig. 3(top). Despite its overt simplicity, both Markov and cryptic orders are infinite. As one should now anticipate, each increase in the length *L* affords a smaller and smaller state entropy, yielding the infinite chain of inequalities: 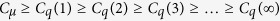. Figure 3(right) verifies this. This sequence approaches the asymptotic value *C*_*q*_(∞) ≃ 1.0332. We also notice that the convergence of *C*_*q*_(*L*) is richer than in the previous processes. For example, while the sequence monotonically decreases (and at each *p*), it is not convex in *L*. For instance, the fourth quantum incremental improvement is greater than the third.

We now turn to discuss the broader theory that underlies the preceding analyses. We first address the convergence properties of *C*_*q*_(*L*), then the importance of studying the full range of memoryful stochastic processes, and finally tradeoffs between synchronization, compression, and prediction.

### *C*_*q*_(*L*) Monotonicity

It is important to point out that while we observed nonincreasing *C*_*q*_(*L*) in our examples, this does not constitute proof. The latter is nontrivial since ref. 27 showed that each pairwise overlap of signal states can increase while *also increasing* von Neumann entropy. (This assumes a constant distribution over signal states.) Furthermore, this phenomenon occurs with nonzero measure. They also provided a criterion that can exclude this somewhat nonintuitive behavior. Specifically, if the element-wise ratio matrix *R* of two Gram matrices of signal states is a positive operator, then strictly increasing overlaps imply a decreasing von Neumann entropy. We note, however, that there exist processes with 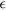-machines for which the *R* matrix is nonpositive. At the same time, we have found no example of an increasing *C*_*q*_(*L*).

So, while it appears that a new criterion is required to settle this issue, the preponderance of numerical evidence suggests that *C*_*q*_(*L*) is indeed monotonically decreasing. In particular, we verified *C*_*q*_(*L*) monotonicity for many processes drawn from the topological 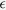-machine library^28^. Examining 1000 random samples of two-symbol, *N*-state processes for 2 ≤ *N* ≤ 7 yielded no counterexamples. Thus, failing a proof, the survey suggests that this is the dominant behavior.

### Infinite Cryptic Order Dominates

**Figure 4:**
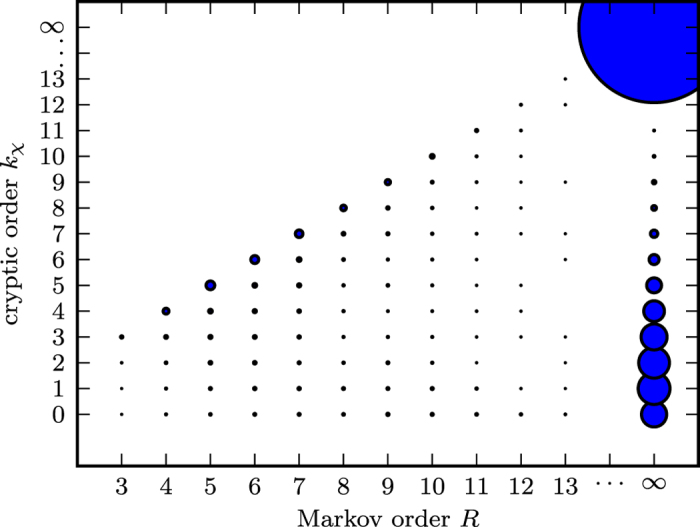
Distribution of Markov order *R* and cryptic order 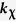 for all 1, 132, 613 six-state, binary-alphabet, exactly-synchronizing 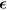-machines. Marker size is proportional to the number of 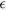-machines within this class at the same (*R*, 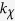). (Reprinted with permission from ref. 29).

The Biased Coins Process, being cryptic order *k* = 1, is atypical. Previous exhaustive surveys demonstrated the ubiquity of infinite Markov and cryptic orders within process space. For example, Fig. 4 shows the distribution of different Markov and cryptic orders for processes generated by six-state, binary-alphabet, exactly-synchronizing 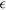-machines^29^. The overwhelming majority have infinite Markov and cryptic orders. Furthermore, among those with finite cryptic order, orders zero and one are not common. Such surveys in combination with the apparent monotonic decrease of *C*_*q*_(*L*) confirm that, when it comes to general claims about compressibility and complexity, it is advantageous to extend analyses to long sequence lengths.

### Prediction-Compression Trade Off

**Figure 5:**
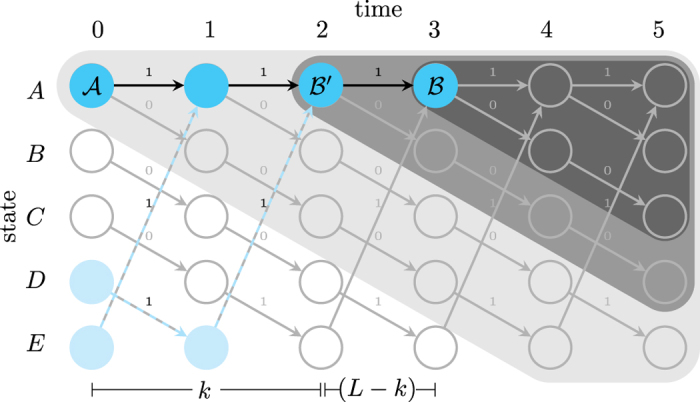
Trading prediction for quantum compression: 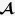 is Alice’s state of predictive knowledge. 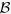
 is that for Bob, except when he uses the process’ 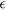-machine to refine it. In which case, his predictive knowledge becomes that in 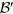, which can occur at a time no earlier than that determined by the cryptic order *k*.

Let’s return to Alice and Bob in their attempt to synchronize on a given stochastic process to explore somewhat subtle trade-offs in compressibility, prediction, and complexity. Figure 5 illustrates the difference in their ability to generate probabilistic predictions about the future given the historical data. There, Alice is in causal state *A* (signified by 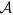 for Alice). Her prediction “cone” is depicted in light gray. It depicts the span over which she can generate probabilistic predictions conditioned on the current causal state (*A*). She chooses to map this classical causal state to a *L* = 3 q-machine state and send it to Bob. (Whether this is part of an ensemble of other such states or not affects the rate of qubit transmission, but not the following argument.) It is important to understand that Bob cannot actually determine the corresponding causal state (at time *t* = 0). He can, however, make a measurement that results in some symbol sequence of length 3 followed by a definite (classical) causal state. In the figure, he generates the sequence 111 followed by causal state *A* at time *t* = 3. This is shown by the blue state-path ending in 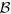 for Bob. Now Bob is in position to generate corresponding *conditional* predictions–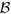
’s future cone 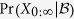 (dark gray). As the figure shows, this cone is only a subprediction of Alice’s. That is, it is equivalent to Alice’s prediction conditioned on her observation of 111 or any other word leading to the same state.

Now, what *can* Bob say about times *t* = 0, 1, 2? The light blue states and dashed edges in the figure show the alternate paths that could have also lead to his measurement of the sequence 111 and state *A*. For instance, Bob can only say that Alice might have been in causal states *A*, *D*, or *E* at time *t* = 0. In short, the quantum representation led to Bob’s uncertainty about the initial state sequence and, in particular, Alice’s prediction. Altogether, we see that the quantum representation gains compressibility at the expense of Bob’s predictive power.

What if Alice does not bother to compute *k* and, wanting to make good use of quantum compressibility, uses an *L* = 1000 q-machine? Does this necessarily translate into Bob’s uncertainty in the first 1000 states and, therefore, only a highly conditional prediction? In our example, Alice was not quite so enthusiastic and settled for the *L* = 3 q-machine. We see that Bob can use his current state *A* at *t* = 3 and knowledge of the word that led to it to infer that the state at *t* = 2 must have been *A*. The figure denotes his knowledge of this state by 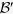. For other words he may be able to trace farther back. (For instance, 000 can be traced back from *D* at *t* = 3 all the way to *A* at *t* = 0.) The situation chosen in the figure illustrates the worst-case scenario for this process where he is able to trace back and discover all but the first 2 states. The worst-case scenario defines the cryptic order *k*, in this case *k* = 2. After this tracing back, Bob is then able to make the improved statement, “If Alice observes symbols 11, then her conditional prediction will be 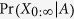
”. This means that Alice and Bob cannot suffer through *overcoding*–using an *L* in excess of *k*.

Finally, one feature that is unaffected by such manipulations is the ability of Alice and Bob to *generate* a single future instance drawn from the distribution 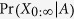. This helps to emphasize that generation is distinct from prediction. Note that this is true for the q-machine *M*(*L*) at any length.

## Methods

Let’s explain the computation of *C*_*q*_(*L*). First, note that the size of the q-machine *M*(*L*) Hilbert space grows as 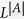 (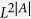 for the density operators). That is, computing *C*_*q*_(*L* = 20) for the Nemo Process involves finding eigenvalues of a matrix with 10^12^ elements. Granted, these matrices are often sparse, but the number of components in each signal state still grows exponentially with the topological entropy rate of the process. This alone would drive computations for even moderately complex processes (described by moderate-sized 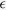-machines) beyond the access of contemporary computers.

Recall though that there are, at any *L*, still only |*S*| quantum signal states to consider. Therefore, the embedding of this constant-sized subspace wastes an exponential amount of the embedding space. We desire a computation of *C*_*q*_(*L*) that is independent of the diverging embedding dimension.

Another source of difficulty is the exponentially increasing number of words with *L*. However, we only need to consider a small subset of these words. Once a merger has occurred between states 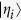 and 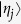 on word *w*, subsequent symbols, while maintaining that merger, do not add to the corresponding overlap. That is, the contribution to the overlap 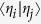 by all words with prefix *w* is complete.

To take advantage of these two opportunities for reduction, we compute *C*_*q*_(*L*) in the following manner.

**Figure 6:**
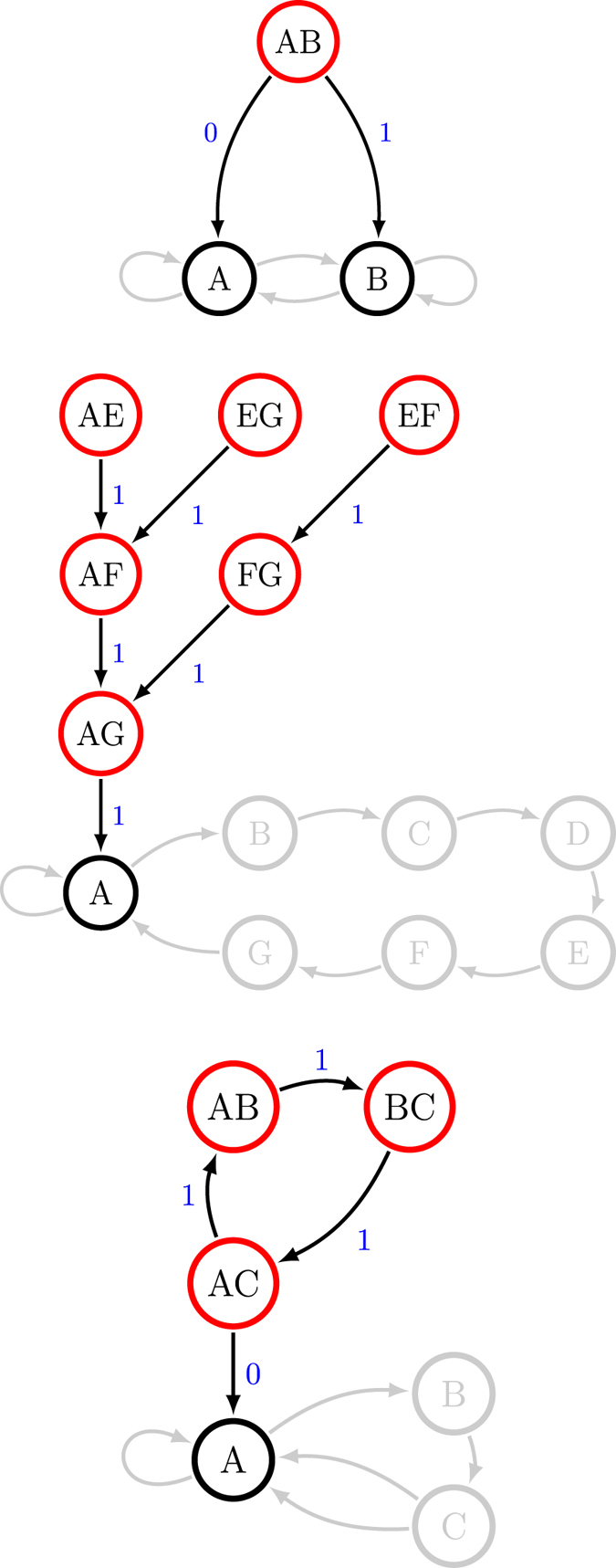
Pairwise-merger machines for our three example processes. Pair-states (red) lead to each other or enter the 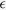-machine at a noncounifilar state. For example, in the R-k Golden Mean (middle), the two pair-states *AF* and *FG* both lead to pair-state *AG* on 0. Then pair-state *AG* leads to state *A*, the only noncounifilar state in this 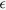-machine.

First, we construct the “pairwise-merger machine” (PMM) from the 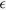-machine. The states of the PMM are unordered pairs of causal states. A pair-state (*σ*_*i*_, *σ*_*j*_) leads to (*σ*_*m*_, *σ*_*n*_) on symbol *x* if *σ*_*i*_ leads to *σ*_*m*_ on *x* and *σ*_*j*_ leads to *σ*_*m*_ on *x*. (Pairs are unordered, so (*σ*_*m*_, *σ*_*n*_) = (*σ*_*n*_, *σ*_*m*_).) If both components in a pair-state lead to the same causal state, then this represents a merger. Of course, these mergers from pair-states occur only when entering noncounifilar states of the 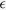-machine. If either component state forbids subsequent emission of symbol *x*, then that edge is omitted. The PMMs for the three example processes are shown in Fig. 6.

Now, making use of the PMM, we begin at each noncounifilar state and proceed backward through the pair-state transient structure. At each horizon-length, we record the pair-states visited and with what probabilities. This allows computing each increment to each overlap. Importantly, by moving *up* the transient structure, we avoid keeping track of any further novel overlaps; they are all “behind us”. Additionally, the finite number of pair-states gives us a finite structure through which to move; when the end of a branch is reached, its contributions cease. It is worth noting that this pair-state transient structure may contain cycles (as it does for the Nemo Process). In that case, the algorithm is non-halting, but it is clear that contributions generated within a cycle decrease exponentially.

All of this serves to yield the set of overlaps at each length. We then use a Gram-Schmidt-like procedure to produce a set of 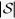 vectors in 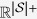 (the positive hyperoctant) having the desired set of overlaps.

Weighting these real, positive vectors with the stationary distribution yields a real, positive-element representation of the density operator restricted to the subspace spanned by the signal states. At this point, computing *C*_*q*_(*L*) reduces to finding eigenvalues of an 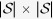 matrix.

For example, consider the Nemo Process. The sequence of overlap *increments* for 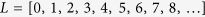, for 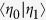, 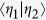, 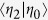 respectively, is given by:


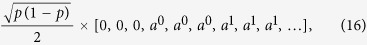



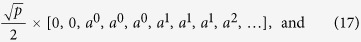



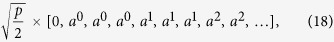


where 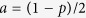.

And, the asymptotic cumulative overlaps are given by:


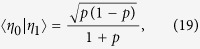



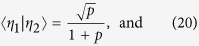



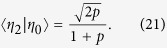


From this, we computed the restricted density matrix and, hence, its *L* → ∞ entropy 
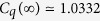
, as illustrated in Fig. 3. The density matrix and eigenvalue forms are long and not particularly illuminating, and so we do not quote them here. A sequel details a yet more efficient analytic technique based on holomorphic functions of the internal-state Markov chain of a related quantum transient structure^30^.

## Discussion

Recalling our original motivation, we return to the concept of *pattern*; in particular, its representation and characterization. We showed that, to stand as a canonical form, a process’ quantum representation should encode, explicitly in its states, process correlations over a sufficiently long horizon-length. In demonstrating this, our examples and analyses found that the q-machine generally offers a more efficient quantum representation than the alternative previously introduced^1^ and, perhaps more importantly, it can be constructed for any process with a finite-state 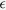-machine.

Interestingly, the length scale at which our construction’s compression saturates is the cryptic order, a recently introduced measure of causal-state merging and synchronization for classical stochastic processes. Cryptic order, in contrast to counifilarity, makes a finer division of process space, suggesting that it is a more appropriate explanation for super-classical compression. We also developed efficient algorithms to compute this ultimate quantum compressibility. Their computational efficiency is especially important for large or infinite cryptic orders, which are known to dominate process space.

We cannot yet establish the minimality of our construction with respect to all alternatives. For example, more general quantum hidden Markov models (QHMMs) may yield a greater advantage^3^. Proving minimality among QHMMs is of great interest on its own, as it would mark a canonical quantum representation of classical stochastic processes. As we have illustrated in Sec. 4, the observed quantum compression has come at a cost–the q-machine is not generally fully predictive (while the 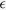-machine is). There exist classical representations that make a similar tradeoff–generative models can be (entropically) smaller than the 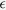-machine, but can only generate instances as opposed to produce full predictive future morphs^31^. Teasing apart the effects of this generative tradeoff from the purely quantum contribution to compression will require a better understanding of classical generative models, itself a nontrivial task. Further, claims about overall minimality of quantum representation will require first defining the appropriate space. We look forward to making contributions toward answering these questions in future work.

And, what is the meaning of the remaining gap between *C*_*q*_(*k*) and **E**? In the case that *C*_*q*_(*k*) is in fact a minimum, this difference should represent a quantum analog of the classical crypticity. Physically, since the latter is connected with information thermodynamic efficiency^22^,^32^,^33^, it would then control the efficiency for quantum thermodynamic processes.

Let’s close by outlining future impacts of these results. Most generally, they provide yet another motivation to move into the quantum domain, beyond cracking secure codes^34^ and efficient database queries^35^. They promise extremely high, super-classical compression of our data. If implementations prove out, they will be valuable for improving communication technologies. However, they will also impact quantum computing itself, especially for Big Data applications, as markedly less information will have to be moved when it is quantum compressed.

## Additional Information

**How to cite this article**: Mahoney, J. R. *et al.* Occam’s Quantum Strop: Synchronizing and Compressing Classical Cryptic Processes via a Quantum Channel. *Sci. Rep.* **6**, 20495; doi: 10.1038/srep20495 (2016).
